# Exploring software navigation tools for liver tumour angiography: a scoping review

**DOI:** 10.1002/jmrs.760

**Published:** 2024-02-02

**Authors:** Nathan Brunskill, John Robinson, Don Nocum, Warren Reed

**Affiliations:** ^1^ San Radiology & Nuclear Medicine Sydney Adventist Hospital Wahroonga New South Wales Australia; ^2^ Sydney School of Health Sciences, Faculty of Medicine and Health University of Sydney Camperdown New South Wales Australia

**Keywords:** 3D imaging, angiography, cancer, cone‐beam computed tomography, interventional oncology, Interventional radiology, liver tumour, medical imaging, medical imaging software, software navigation

## Abstract

**Introduction:**

Liver cancer presents a growing global health concern, necessitating advanced approaches for intervention. This review investigates the use and effectiveness of software navigation in interventional radiology for liver tumour procedures.

**Methods:**

In accordance with Preferred Reporting Items for Systematic reviews and Meta‐Analyses extension for Scoping Reviews (PRISMA‐ScR) guidelines, a scoping review was conducted of the literature published between 2013 and 2023 sourcing articles through MEDLINE, Scopus, CINAHL and Embase. Eligible studies focused on liver cancer, utilised cone‐beam computed tomography (CBCT), and employed software for intervention. Twenty‐one articles were deemed eligible for data extraction and analysis.

**Results:**

Categorised by type, software applications yielded diverse benefits. Feeder detection software significantly enhanced vessel identification, reducing non‐target embolisation by up to 43%. Motion correction software demonstrated a 20% enhancement in image quality, effectively mitigating breathing‐induced motion artefacts. Liver perfusion software facilitated efficient tumour targeting while simultaneously reducing the occurrence of side effects. Needle guide software enabled precise radiofrequency ablation needle placement. Additionally, these software applications provided detailed anatomical simulations. Overall, software integration resulted in shorter procedures, reduced radiation exposure and decreased contrast media usage.

**Conclusion:**

This scoping review highlights the innovative yet relatively underexplored role of software navigation for liver tumour procedures. The integration of software applications not only enhances procedural efficiency but also bolsters operator confidence, and contributes to improved patient outcomes. Despite the current lack of uniformity and standardisation, these software‐driven advancements hold significant promise for transforming liver tumour interventions. To realise these benefits, further research is needed to explore the clinical impact and optimal utilisation of software navigation tools in interventional radiology.

## Graphical abstract

The paper discusses the use of advanced software navigation in interventional oncology for liver cancer treatment, focusing on its potential benefits, such as increased precision and reduced radiation exposure. Through a scoping review of 21 studies, it highlights how software‐guided interventions can improve precision, reduce procedural time, lower radiation exposure and minimise contrast media usage. The paper concludes that integrating software navigation into angiography procedures holds promise for enhancing liver cancer treatment, provided that appropriate training and adaptation of practices occur.

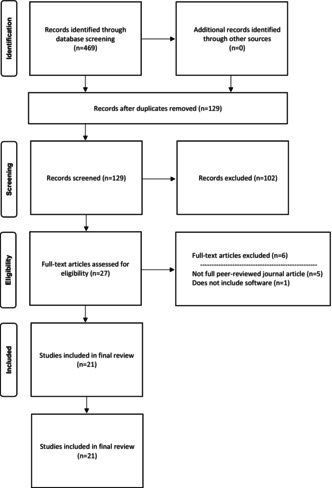

## Introduction

Globally, approximately 10 million deaths were attributed to cancer in 2020, marking an increase from 5.7 million in 1990.^1^ Among these cancer cases, liver cancer accounted for approximately 830,000 deaths, an approximate increase of approximately 457,000 cases in this timeframe.^2^ Although the number of cases has risen, when analysed versus the population rise, the number of deaths has begun to fall by 2.98 liver cancer deaths per 100,000 people worldwide.^1^ With the advances in imaging and interventional oncology (IO) it would be reasonable to credit these as contributing to the reduction in deaths. Initially conceived as a minimally invasive, image‐guided tumour therapy, IO has become a comprehensive and innovative specialty driving new technological developments. These advancements enable physicians to administer therapeutics with greater precision, specifically targeting tumours while minimising damage to healthy tissue.^3^, ^4^ Exemplar procedures include transcatheter arterial chemoembolisation (TACE) and selective internal radiation therapy (SIRT). One significant development in this field is the integration of cone beam computed tomography (CBCT) with various software programs, which facilitates treatment planning and delivery of chemotherapy and/or radiation therapy agents. CBCT provides a three‐dimensional (3D) visualisation of tumour‐feeding arteries and can identify lesions that may not be visible on digital subtraction angiography (DSA).^5^ Traditionally, the assessment of intraoperative tumours typically relies on the conventional technique of two‐dimensional (2D) angiography. This can be limited for arterial assessment especially if hypervascular tumours are present. If feeder vessels are extremely small calibre then DSA alone will not be able to identify them. However, by aligning two imaging datasets spatially, CBCT imaging allows for real time visualisation of anatomical structures by overlaying software‐enhanced volume‐rendered images onto live fluoroscopy. This integration enables easy navigation and visualisation of tumour vessels, guiding operators to target arteries accurately using live image guidance.

Current literature describing software utilisation in practice for liver tumour angiography procedures is sparse, and the predominant published papers only provide descriptive definitions of the software. These papers do offer insights into the capabilities of the software, emphasising its role as an advanced 3D clinical analysis tool.^6^, ^7^ However, there is a lack of practical investigation into concepts such as potential reductions in radiation dose and iodinated contrast usage to reach a feasible consensus.^8^ Firstly, the high cost associated with the equipment required to perform these procedures poses a barrier. Secondly, concerns regarding patient radiation overexposure, resulting from the combination of two imaging modalities that each deliver high doses of radiation have contributed to limited exploration in the area.^9^

Recent research has shown a growing interest into the application of CBCT and 3D imaging for liver interventions. However, there is limited research specifically examining the use and effectiveness of 3D guidance software for oncological liver procedures. Therefore, the purpose of this scoping review was to assess the effectiveness of software navigation in liver tumour procedures.

## Methodology

### Method of review

The review methodology followed the predetermined guidelines outlined in the Preferred Reporting Items for Systematic Reviews and Meta‐Analyses – Scoping review extension (PRISMA‐ScR) framework.

### Identifying the relevant studies

The databases searched for relevant literature included MEDLINE, Scopus, Cumulative Index to Nursing and Allied Health Literature (CINAHL) and Embase. The search strategy was divided into two sections. The first section focused on the population (Liver Cancer) and context (Visualisation) aspects of the search construction. The second section addressed the concept (Software) aspect, with each term combined with the initial search results. All relevant Medical Subject Heading (MeSH) terms and CINAHL subject headings were included as listed in Table 1. The complete search terms can be found in Appendix S1.

**Table 1:** Keywords and medical subject heading search terms.

	Construction method	Search term	
Initial Search	Population (Liver Cancer)	Hepatocellular Carcinoma. *OR* Carcinoma, Hepatocellular/ *OR* Hepatobiliary tumo?r(s). *OR* Hepatobiliary cancer(s). *OR* Liver cell carcinoma(s). *OR* Liver cancer(s). *OR* Fibrolamellar hepatocellular carcinoma(s).	
		*AND*	
	Context (Visualisation)	Cone‐beam CT. *OR* Cone‐Beam Computed Tomography/ *OR* CBCT. *AND* Angiography. *OR* Angiography, Digital Subtraction/ *OR*Angiography/	
		*AND*	
	Language	*Limit to English*	
Secondary Search	Concept (Software)	*1*	Software. *OR* Software/
		*2*	Virtual Reality/ *OR* Virtual.
		*3*	Spatial Navigation/ *OR* Navigation. *OR* Patient Navigation/ *OR* Surgical Navigation Systems/
		*4*	Imaging, Three‐Dimensional/ *OR* Three Dimensional.mp. *OR* Image Processing, Computer‐Assisted/ *AND* 3D.
		*5*	Analysis.
		*6*	Artificial Intelligence. *OR* Artificial Intelligence/

Articles were considered eligible if they focused on liver cancer and employed DSA and CBCT as methods of visualisation, utilising software and navigation technology for data interpretation. The initial literature search spanned from 01 January 2006 to 01 February 2023, and only full‐text, peer‐reviewed studies written in English were accepted. Any studies that did not meet these criteria were excluded. Given the recent nature of the topic, most of the sources identified were contemporary publications, with the earliest being published in 2013.

### Study selection

**Figure 1:**
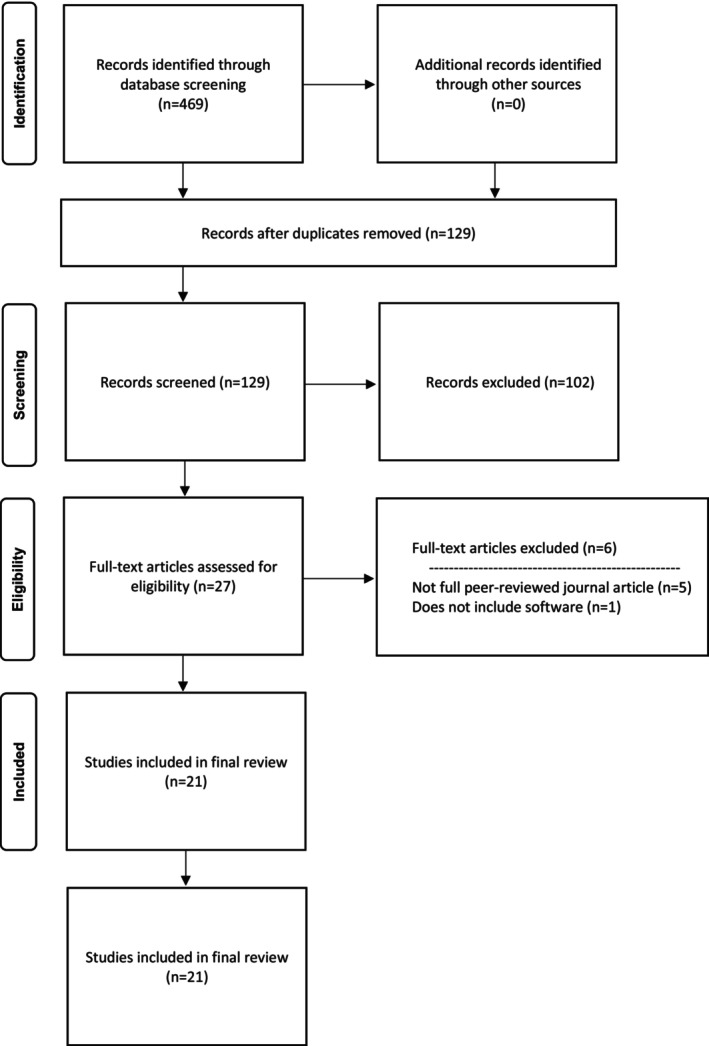
PRISMA flow diagram.

After conducting a database search, a total of 469 results were obtained. These studies were organised in Endnote™ X9 (California, United States) and then imported into Covidence (Victoria, Australia), an online reference management system and systematic review software.^10^ Through an automated process, 340 articles were removed as duplicates, leaving 129 articles to be screened. Three reviewers (NB, WR, JR) independently reviewed the titles and abstracts of these articles, resulting in the exclusion of a further 102 articles. The reviewers then conducted a full text review and reached a consensus on acceptance, leading to the removal of an additional six articles; 21 articles remained for data extraction. This entire process is visually presented in the PRISMA flow diagram, as shown in Figure 1.

### Charting the data

A charting table was created and populated with relevant data from each paper. The charted data included key characteristics and findings of the studies, such as the study purpose and design, number of subjects, equipment and software used, study findings and conclusions drawn.

The data sources contained variables related to the participants, study design, number of participants, equipment and software used, the number and experience of readers and the evaluated procedure. The results reported in the studies varied, which could be attributed to the chronological span of studies. The studies were conducted over an 8‐year period, with advancements in software and hardware technologies in the field, any comparisons between studies need to be considered with this caveat in mind. The included studies comprised a combination of prospective and retrospective designs, utilising various software programs and applications. The analysis included all types of software used, documenting any novel or previously unreported uses of the technology.

### Collating, summarising and reporting results

The data extracted from tables were categorised and synthesised using a thematic analysis approach. The studies were grouped based on the types and uses of software and subjected to methodological rigour to enhance credibility and accuracy.

## Results

The search yielded results from 2013 to 2023. Of the 21 articles, the majority (*n* = 12) were published after 2018 indicating a growing interest in software usage and its capabilities for angiographic applications. These studies were categorised into three types: retrospective (*n* = 13), prospective (*n* = 6) and the remaining two articles were unclassified. Among the defined studies, most were comparative (*n* = 13), while the others were case studies (*n* = 6). The primary procedure studied was TACE (*n* = 16). Other procedures observed included transarterial embolisation (*n* = 2) and SIRT (*n* = 1). Regarding software programs used in these studies, FlightPlan for Liver® (GE Healthcare, Waukesha, Wisconsin) and EmboGuide® (Philips Healthcare, Best, The Netherlands) were the most frequently used, with six inclusions each. All other studies either described a single software program or a non‐commercial prototype software.

The charting process led to the categorisation of software types into five main themes: feeder detection software, motion correction software, liver perfusion software, needle guide software, and unique software applications. The primary application of the software determined the thematic selection. In cases where studies involved software with multiple applications, the theme that was most predominantly aligned with the software's main purpose was chosen.

### Feeder detection software

A total of 12 studies were reported on feeder detection software.^11^, ^12^, ^13^, ^14^, ^15^, ^16^, ^17^, ^18^, ^19^, ^20^, ^21^, ^22^ This visualisation and guidance software utilises a CBCT dataset to distinguish and define all vessels from the imaging source to the tumour via contrast‐enhanced arteries, with the goal to avoid non‐target embolisation. When compared to conventional DSA the software demonstrated a significant improvement in accurately detecting feeding arteries by 43%.^17^, ^18^, ^21^, ^22^ Three papers described it as more sensitive compared to conventional CBCT and DSA.^11^, ^12^, ^15^ Moreover, it detected additional tumours not identified on pre‐procedural cross‐sectional imaging.^15^, ^19^, ^22^ Eight papers noted significant decreases in standard markers, including procedural time and radiation exposure,^15^, ^21^ the number of angiographic acquisitions^12^, ^13^, ^14^, ^20^, ^21^ and contrast medium usage.^17^, ^21^ The time‐saving benefit of this technology is widely documented^14^, ^15^, ^17^, ^20^, ^21^ and is described as rapid and reliable.^15^

### Motion correction software

Two studies examined the feasibility of using software enhancement to correct artefacts resulting from breathing motion.^23^, ^24^ CBCT imaging is affected by respiratory motion artefacts, which were reported to corrupt image quality and reduce diagnostic confidence in up to 10% of cases.^23^ Both studies described an increase in image quality with no significant degradation.^23^ The software improved image quality in 88% of cases and increased structure visibility in 75% of cases.^23^ The application of the motion correction algorithm resulted in a 20% increase in image sharpness,^24^ with image quality degradation reported in only 3% of cases.^23^

### Liver perfusion software

Two retrospective studies investigated software to calculate liver perfusion.^25^, ^26^ One was a comparative study, while the other was a case study. One study explored software for procedure planning,^25^ while the other assessed the effectiveness of the post‐procedural treatment in terms of technical success and treatment response.^26^ The software allowed tumour vascularity to be displayed in vivid colours based on the perfusion degree.^26^ When multiple tumour feeders were present, they were colour‐coded separately.^25^

The use of this software enhanced progress by identifying viable liver masses, leading to increased therapeutic effects, reduced side effects, and the preservation of liver reserve. This was achieved through more accurate tumour targeting, reducing non‐target embolisation.^26^ Kinoshita et al. documented that the software allowed for confident determination of the optimal catheter position, including the tumour and the safety margin for embolisation.^25^ When used in conjunction with TACE, it increased arterial enhancing tissue corresponding to residual tumours, improving treatment efficiency and reducing the risk of overlooking small residual tumours.^26^

### Needle guide software

Needle guidance software was discussed in two papers. CBCT images were utilised to determine the tumour's location, enabling the operator to plan a safe path for the needle to navigate through anatomical structures to reach the desired location. Yamada et al. and Yao et al. used the software to assist in treating tumours via radiofrequency ablation (RFA). Yamada et al. observed a decrease in the average number of cross‐sectional image acquisitions compared to the conventional method of CT alone, resulting in a lower median radiation dose.^27^ Yao et al. found that the median dose area product (DAP) of the procedures was consistent with findings reported in previous literature. The treatment response post‐RFA strongly aligned with the pre‐RFA planning estimation, demonstrating high accuracy and statistical significance. This led to reduced operation time and fewer therapy‐associated complications.^28^ Both papers achieved a 100% technical success rate.^27^, ^28^

### Unique software applications

Three papers could not be categorised into themes of a similar nature, as their use of software was unique. Each paper either used the software in a novel manner or examined the impact of software imaging systems for intervention, which could not be compared to other reported practices. Due to the uniqueness of these studies within this theme, no comparative data could be derived from them.

Schernthaner et al. conducted a prospective comparative study comparing older versus newer technology, resulting in a significant reduction in radiation exposure of up to 66% for digital fluoroscopy (DF) and DSA. The DSA runs showed the highest radiation exposure reduction (84%). The exposure during DF and CBCT runs decreased by 47% and 15% respectively.^29^ There was no statistically significant difference reported regarding image quality.

A single patient report by Mohammed et al. described the potential role of interactive virtual reality (VR) within the context of interventional radiology.^30^ VR offered more precise vessel diameter and length measurements, especially for tortuous arteries.^30^ However, VR has limitations due to its lack of temporal resolution and limited in‐plane resolution.^30^

Simoncini et al. proposed generating a patient‐specific simulation of the entire hepatic arterial tree via a computational model based on the segmentation of a patient's tomography.^31^ This approach achieved good correspondence between real and in silico arterial trees, depicting down to arterioles with a diameter of ∼0.05 mm. It provided a preliminary simulation of microspheres injection with quantification of their distribution in tumour and healthy tissue.^31^

## Discussion

This scoping review examined 21 papers that described the use of CBCT software programs for interventional radiology applications. Despite the variation in study depth and software exploration, one common thread emerged: the developments in this field empower operators to provide more accurate and decisive treatment to patients. While these software applications may not be widely researched and utilised, the published literature demonstrates clear advantages compared to more conventional techniques. Instead of focusing solely on the various uses of the software, it is more valuable in a radiographic context, to examine the overall benefits software offers to the healthcare environment. Recognising these overall advantages is crucial for integrating the software into becoming best practice for the treatment of liver cancers.

Time is a precious commodity in healthcare, and software that reduces procedural time, offers significant benefits. Multiple studies have demonstrated that the software has led to faster procedures.^14^, ^17^, ^19^, ^23^, ^28^ Iwazawa et al. reported that the software contributed to procedures being completed approximately 12% more quickly.^14^ Specific software features facilitate the acceleration of certain parts of the procedure. Multifunctional 3D roadmaps aid operators in positioning catheters more swiftly.^17^ Motion correction software enhances CBCT image quality, preventing unnecessary retakes and decreasing procedural time.^23^ Feeder detection software operates quickly, also reducing procedural time.^14^ However, some applications take a considerable amount of time to operate successfully. For example, the virtual liver parenchymal perfusion area simulations take 30 minutes to generate.^25^ While useful, this may be too time consuming for busy operators. Nevertheless, the software allows operators to reduce DSA and fluoroscopy burdens, expediting procedure time. The inclusion of CBCT and software manipulation increases workload and subsequently extends time in image manipulation.^20^ Nevertheless, this additional effort reduces time for the remainder of the procedure due to the overlay technology it provides to the operator. Additionally, 3D roadmaps enable quicker visualisation and navigation of vessels.^17^, ^19^, ^21^

The introduction of software applications is crucial because it provides operators navigating extremely complex anatomy with more detailed images, information and models from which to base treatment decisions. Enhanced vessel visibility improves the demarcation of vessels from surrounding structures, leading to a reduction of artefacts by foreign material.^24^ The software accurately detects blood vessels,^14^, ^15^, ^17^, ^18^ avoiding unnecessary catheterisation of non‐target branches.^13^ However, the software has limitations. It can struggle with hyper vascular tumour nodules, and very fine distal‐feeding arteries, and target definition can be challenging in irregular shaped tumours or those adjacent to other organs, potentially leading to misinterpretation.^22^ Sensitivity, while improved, is not 100%.^12^ Software applications boost clinical confidence by increasing treatment accuracy. Needle guidance provides immediate feedback on treatment progression by illustrating a safe path for directing an ablation needle into a tumour, enabling the assessment of ablated areas within that tumour.^27^, ^28^ Liver perfusion mapping aids in identifying a viable liver mass, reducing side effects, and preserving liver reserve. This precision in targeting tumours reduces non‐target embolisation and improves patient prognosis,^26^ particularly when used with TACE, reducing the risk of overlooking small residual tumours.^26^

Radiation dose is a paramount concern in interventional procedures, and the objective is to maintain the radiation dose as low as reasonably achievable (ALARA) while ensuring quality treatment. The introduction of software applications must not compromise this fundamental ALARA principle. This aspect was well addressed by the studies, showing that, at the very least, the radiation dose was comparable to conventional techniques,^27^ or even reduced.^17^, ^27^, ^29^ There was a paucity of dose information in the articles and only two studies included a comparison of patient dose between pre and post software which is highlighted in Table 2. Even if not quantified directly, many studies indicated that CBCT and software reduced the need for fluoroscopy and DSA runs,^12^, ^14^, ^18^, ^20^, ^23^ leading to reduced radiation dose. There is a compelling suggestion that the rotational nature of the CBCT image acquisition and the resulting rotating x‐ray exposure to the patient can mitigate radiation‐induced skin injuries and long‐term risks.^20^ Furthermore, the increased utilisation of CBCT imaging over DSA allows operators to reduce their own radiation exposure, as this can be conducted from within the control room.^20^ 3D roadmaps enable the visualisation and navigation of vessels with a reduced dose.^17^, ^21^ The ability to overlay onto live fluoroscopy offers optimal projection angles for catheterisation that can be pre‐determined without further radiation use,^20^ and facilitates ultra‐selective access for all tumours.^15^, ^22^

**Table 2:** Radiation dose summary with and without software.

Author	Dose without software (Median effective dose)	Dose with software (Median effective dose)
Yamada et al. (2018)^27^	68.8 mSv (range 49.75–168.51)	55.4 mSv (range 13.9–253.39)
Schernthaner et al. (2015)^29^	(Gy*cm^2^) 395.8 (range 86.2–1469.9)	(Gy*cm^2^) 132.9 (range 30.7–588.4)

The contribution of contrast media to kidney injury is widely acknowledged. Within angiographic procedures, contrast‐associated acute kidney injury (CA‐AKI) has an incidence rate ranging from three and 37%.^32^ Reducing contrast usage is desirable for improved patient outcomes. Software generated 3D roadmaps facilitate the visualisation and navigation of vessels in less time, leading to a reduction in the required amount of contrast medium.^21^, ^23^ Software usage can prevent unnecessary additional CBCT retakes, consequently decreasing contrast volume.^23^ Software usage has been shown to enhance vessel detection, leading to enhanced operator confidence and subsequently reduced contrast media volumes.^13^ The software provides the operator with enhanced image quality and shorter procedural times. These factors collectively contribute to a decreased requirement for contrast media, thereby reducing the risk of patients experiencing CA‐AKI, a condition for which no treatment exists.

### Limitations

The absence of uniformity and standardisation in the use of software applications for angiographic procedures stands out as a notable aspect. This represents a potential limitation of this scoping review. Numerous papers focus on prototypes or novel software applications that are not commercially available, which somewhat diminishes the applicability of the reported data.

Another potential limitation lies in this review's exclusive focus on software related to liver interventions. The inclusion of software applications aimed at other angiographic procedures may have facilitated more comprehensive data interpretation. Also noted was that among the gathered papers, no critical appraisal was conducted as this is beyond the scope of a scoping review. Consequently, the absence of potential high‐quality evidence could potentially undermine the robustness of the conclusions drawn for this review.

## Conclusion

This scoping review highlights the limited amount of research in contrast to the innovative and widespread use of software applications within interventional radiology. Those willing to embrace these systems should be encouraged to do so and approach it by adapting current practices and providing adequate staff training to maximise its potential. The integration of software applications into the angiography suite offers a host of advantages, including quicker procedures with reduced radiation and contrast media doses, benefiting both patients and the procedural team. Additionally, the improved quality of software‐manipulated image data aids operators in making real‐time clinical decisions. Further research into the clinical impact of software navigation tools for optimising practice in interventional radiology is strongly recommended.

## Conflict of Interest

The authors declare no conflict of interest.

## Supporting information


**Appendix S1** Tabulated search strategy terms and results.

## Data Availability

Data sharing not applicable to this article as no datasets were generated or analysed during the current study.
