# Supplementary material for: Exploring software navigation tools for liver tumour angiography: a scoping review
Source: J Med Radiat Sci. 2024 Feb 2;71(2):261–8. doi: 10.1002/jmrs.760 (PMC11177025; doi:10.1002/jmrs.760)
Supplement: Supplementary file 1 — Appendix S1 Tabulated search strategy terms and results. [file JMRS-71-261-s001.docx]

# Appendix

*Appendix A: Tabulated search strategy terms and results.*

## Scopus

| ***Database*** | ***Term Number*** | ***Term*** | ***Or*** | ***And*** | ***Results*** |
| --- | --- | --- | --- | --- | --- |
| *Scopus* | 1 | "Hepatocellular Carcinoma" OR  "hepatobiliary cancer*" OR  "hepatobiliary tumor*" OR  "hepatobiliary tomour*" |  |  | 110,328 |
|  | 2 | "Liver cell carcinoma*" OR "Liver cancer*" |  |  | 161,376 |
|  | 3 | "Fibrolamellar hepatocellular carcinoma*" |  |  | 433 |
|  | 4 |  | 1,2,3 |  | 186,157 |
|  | 5 | *"Cone-beam CT" OR*  *"Cone-Beam Computed Tomography"* |  |  | 22,633 |
|  | 6 | *"CBCT"* |  |  | 13,096 |
|  | 7 |  | 5,6 |  | 24,141 |
|  | 8 | "Digital Subtraction Angiography" OR  "Angiography" |  |  | 396,045 |
|  | 9 |  |  | 4,7,8 | 131 |
|  | 10 | Limit 9 to English |  |  | 150 |
|  | 11 | "Software" |  |  | 1,502,172 |
|  | 12 |  |  | 10,11 | 30 |
|  | 13 | "Virtual" OR  "Virtual Reality" |  |  | 465,318 |
|  | 14 |  |  | 10,13 | 5 |
|  | 15 | "Navigation" OR  "Spatial Navigation" OR  "Patient Navigation" OR  "Surgical Navigation Systems" |  |  | 229,350 |
|  | 16 |  |  | 10,15 | 6 |
|  | 17 | Three-Dimensional "Three Dimensional Imaging" OR "Computer-Assisted Image Processing" |  |  | 112,073 |
|  | 18 | "3D" |  |  | 784,082 |
|  | 19 |  | 17,18 |  | 849,750 |
|  | 20 |  |  | 10,19 | 29 |
|  | 21 | "Analysis" |  |  | 17,487,618 |
|  | 22 |  |  | 10,21 | 69 |
|  | 23 | "Artificial Intelligence" |  |  | 375,665 |
|  | 24 |  |  | 10,23 | 0 |

## Medline

| ***Database*** | ***Term Number*** | ***Term*** | ***Or*** | ***And*** | ***Results*** |
| --- | --- | --- | --- | --- | --- |
| *Medline* | 1 | Hepatocellular Carcinoma.mp. or Carcinoma, Hepatocellular/ |  |  | 103600 |
|  | 2 | Hepatobiliary tumo?r*.mp. |  |  | 120 |
|  | 3 | Hepatobiliary cancer*.mp. |  |  | 258 |
|  | 4 | Liver cell carcinoma*.mp. |  |  | 336 |
|  | 5 | Liver cancer*.mp. |  |  | 18431 |
|  | 6 | Fibrolamellar hepatocellular carcinoma*.mp. |  |  | 311 |
|  | 7 |  | 1,2,3,4,5,6 |  | 113330 |
|  | 8 | Cone-beam CT.mp. or Cone-Beam Computed Tomography/ |  |  | 11569 |
|  | 9 | CBCT.mp. |  |  | 7351 |
|  | 10 |  | 8,9 |  | 12428 |
|  | 11 | Angiography.mp. or Angiography, Digital Subtraction/ or Angiography/ |  |  | 269441 |
|  | 12 |  |  | 7,10,11 | 48 |
|  | 13 | Limit 12 to English |  |  | 55 |
|  | 14 | Software.mp. or Software/ |  |  | 217818 |
|  | 15 |  |  | 13,14 | 11 |
|  | 16 | Virtual Reality/ or Virtual.mp. |  |  | 49808 |
|  | 17 |  |  | 13,16 | 2 |
|  | 18 | Spatial Navigation/ or Navigation.mp. or Patient Navigation/ or Surgical Navigation Systems/ |  |  | 20354 |
|  | 19 |  |  | 13,18 | 2 |
|  | 20 | Imaging, Three-Dimensional/ or Three Dimensional.mp. or Image Processing, Computer-Assisted/ |  |  | 304231 |
|  | 21 | 3D.mp. |  |  | 119127 |
|  | 22 |  | 20,21 |  | 361972 |
|  | 23 |  |  | 13,22 | 13 |
|  | 24 | Analysis.mp. |  |  | 5978118 |
|  | 25 |  |  | 13,24 | 15 |
|  | 26 | Artificial Intelligence.mp. or Artificial Intelligence/ |  |  | 27446 |
|  | 27 |  |  | 13,26 | 0 |

## Embase

| ***Database*** | ***Term Number*** | ***Term*** | ***Or*** | ***And*** | ***Results*** |
| --- | --- | --- | --- | --- | --- |
| *Embase* | 1 | Hepatocellular Carcinoma.mp. or Carcinoma, Hepatocellular/ |  |  | 186027 |
|  | 2 | Hepatobiliary tumo?r*.mp. |  |  | 210 |
|  | 3 | Hepatobiliary cancer*.mp. |  |  | 579 |
|  | 4 | Liver cell carcinoma*.mp. |  |  | 164931 |
|  | 5 | Liver cancer*.mp. |  |  | 54526 |
|  | 6 | Fibrolamellar hepatocellular carcinoma*.mp. |  |  | 549 |
|  | 7 |  | 1,2,3,4,5,6 |  | 220473 |
|  | 8 | Cone-beam CT.mp. or Cone-Beam Computed Tomography/ |  |  | 21432 |
|  | 9 | CBCT.mp. |  |  | 14261 |
|  | 10 |  | 8,9 |  | 24605 |
|  | 11 | Angiography.mp. or Angiography, Digital Subtraction/ or Angiography/ |  |  | 365619 |
|  | 12 |  |  | 7,10,11 | 143 |
|  | 13 | Limit 12 to English |  |  | 153 |
|  | 14 | Software.mp. or Software/ |  |  | 384536 |
|  | 15 |  |  | 13,14 | 33 |
|  | 16 | Virtual Reality/ or Virtual.mp. |  |  | 90771 |
|  | 17 |  |  | 13,16 | 7 |
|  | 18 | Spatial Navigation/ or Navigation.mp. or Patient Navigation/ or Surgical Navigation Systems/ |  |  | 206841 |
|  | 19 |  |  | 13,18 | 7 |
|  | 20 | Imaging, Three-Dimensional/ or Three Dimensional.mp. or Image Processing, Computer-Assisted/ |  |  | 340996 |
|  | 21 | 3D.mp. |  |  | 238414 |
|  | 22 |  | 20,21 |  | 480672 |
|  | 23 |  |  | 13,22 | 34 |
|  | 24 | Analysis.mp. |  |  | 9416540 |
|  | 25 |  |  | 13,24 | 67 |
|  | 26 | Artificial Intelligence.mp. or Artificial Intelligence/ |  |  | 33693 |
|  | 27 |  |  | 13,26 | 0 |

## CINAHL

| ***Database*** | ***Term Number*** | ***Term*** | ***Or*** | ***And*** | ***Results*** |
| --- | --- | --- | --- | --- | --- |
| *CINAHL* | 1 | "hepatocellular carcinoma" OR (MH "Carcinoma, Hepatocellular") |  |  | 14,800 |
|  | 2 | "hepatobiliary tum*" |  |  | 18 |
|  | 3 | "Hepatobiliary cancer*" |  |  | 74 |
|  | 4 | "Liver cell carcinoma*" |  |  | 9,308 |
|  | 5 | "liver cancer*" |  |  | 17,025 |
|  | 6 | "Fibrolamellar hepatocellular carcinoma*" |  |  | 43 |
|  | 7 |  | 1,2,3,4,5,6 |  | 21,814 |
|  | 8 | cone-beam CT |  |  | 1,028 |
|  | 9 | "cone beam computed tomography" |  |  | 3,235 |
|  | 10 | "CBCT" |  |  | 2,741 |
|  | 11 |  | 8,9,10 |  | 4,485 |
|  | 12 | (MH "Angiography+") OR "Angiography" OR (MH "Angiography, Digital Subtraction") |  |  | 60,505 |
|  | 13 |  |  | 7,11,12 | 38 |
|  | 14 | Limit 13 to English |  |  | 44 |
|  | 15 | (MH "Software+") OR "Software" |  |  | 424,160 |
|  | 16 |  |  | 14,15 | 14 |
|  | 17 | (MH "Virtual Reality+") OR "Virtual" |  |  | 23,078 |
|  | 18 |  |  | 14,17 | 2 |
|  | 19 | (MH "Patient Navigation") OR "Navigation" |  |  | 7,707 |
|  | 20 | "Spatial Navigation" OR (MH "Spatial Perception") |  |  | 3,438 |
|  | 21 | "Surgical Navigation Systems" |  |  | 20 |
|  | 22 |  | 19,20,21 |  | 10,929 |
|  | 23 |  |  | 14,22 | 2 |
|  | 24 | (MH "Imaging, Three-Dimensional+") OR "Three Dimensional" |  |  | 28,721 |
|  | 25 | (MH "Image Processing, Computer Assisted+") OR "Computer-assisted image processing" |  |  | 40,646 |
|  | 26 | "3D" |  |  | 17,305 |
|  | 27 |  | 24,25,26 |  | 66,097 |
|  | 28 |  |  | 14,27 | 15 |
|  | 29 | "Analysis" |  |  | 1,496,082 |
|  | 30 |  |  | 14,29 | 15 |
|  | 31 | "Artificial Intelligence" |  |  | 6,616 |
|  | 32 |  |  | 14,31 | 0 |
